# Controlling Transmission of MRSA to Humans During Short-Term Visits to Swine Farms Using Dust Masks

**DOI:** 10.3389/fmicb.2018.03361

**Published:** 2019-01-17

**Authors:** Øystein Angen, Lotte Skade, Tinna Ravnholt Urth, Mikael Andersson, Poul Bækbo, Anders Rhod Larsen

**Affiliations:** ^1^Department of Bacteria, Parasites and Fungi, Statens Serum Institut, Copenhagen, Denmark; ^2^SEGES Danish Pig Research Centre, Kjellerup, Denmark; ^3^Department of Epidemiology Research, Statens Serum Institut, Copenhagen, Denmark

**Keywords:** MRSA, dust mask, swine farm, protection, contamination dose

## Abstract

The aim of the investigation was to determine the protective effect of wearing dust masks to prevent Methicillin-resistant *Staphylococcus aureus* (MRSA) contamination during a short-term visit in a MRSA-positive swine farm. A total of 118 human volunteers were randomly allocated to a group wearing masks and to the control group. On average, 9% of the participants wearing masks were MRSA-positive when leaving the farm compared to 62% of the participants not wearing masks. At day 1, 2, and 7 after the farm visit, none of those wearing masks were MRSA-positive. An odds ratio of 18.9 (CI: 6.4–56.2) for being MRSA-positive was found for those not wearing masks compared to those wearing masks when including the farm as random effect. None of the covariates (gender, age, and smoking) influenced the OR for being MRSA-positive when leaving the farm. This study shows that the use of dust masks gives a significant protection against nasal MRSA contamination after a short-term visit to a swine farm. In addition, 106 volunteers not wearing masks were investigated in order to provide an estimate of a contamination dose of airborne MRSA. Due to the high variation in the MRSA measurements, it was not possible to establish a stable estimate for this. Out of the 106 volunteers not wearing masks, 6, 7, and 3% tested MRSA-positive 1, 2, and 7 days after the farm visit, respectively.

## Introduction

Methicillin-resistant *Staphylococcus aureus* (MRSA), are resistant to most beta-lactam antibiotics and has primarily been associated to hospitals but have since the 1990s increasingly been found in the community outside hospitals. In 2005, MRSA belonging to clonal complex 398 (CC398) was first described in pigs and pig farmers in France and the Netherlands (Armand-Lefevre et al., 2005; Voss et al., 2005). MRSA CC398 has later been disseminated in the pig production worldwide, but as it is also found in other livestock animals (Graveland et al., 2010; van Duijkeren et al., 2016), it is commonly designated livestock-associated MRSA (LA-MRSA).

Transmission of LA-MRSA from animals to humans has been of great concern in some European countries, especially those with low MRSA incidence and large pig productions. In concordance with the increasing level of LA-MRSA positive Danish swine herds reaching 88% in 2016 (DANMAP, 2016), a steep increase in human LA-MRSA cases in Denmark has been registered. This has primarily been in persons with contact to swine farms, however, 33% of LA-MRSA infections in Denmark are not associated with livestock contact (Larsen et al., 2015). Transmission studies have shown that humans are transiently contaminated by LA-MRSA after a short-term visit to a swine farm, that this is correlated to the amount of airborne MRSA, but also that the level of contamination declines rapidly to unquantifiable levels already after a few hours (Angen et al., 2017).

In order to diminish spread to the general public, attempts to restrict LA-MRSA to the farms have been made, e.g., by improved hygiene routines for farm workers. Staphylococci have been shown to be associated with dust particles (Zhao et al., 2017; Feld et al., 2018) and during a visit to a swine farm dust particles will be deposited on the skin and the nasal mucosa. Nathaus et al. (2011) showed that four out of five veterinarians wearing dust masks were protected against MRSA colonization during a study period of 30 days.

In the present investigation, the protective effect of wearing dust mask (FFP2D-masks) for short time visitors was evaluated and an attempt was made to obtain an estimate of a contamination dose of airborne MRSA.

## Materials and Methods

### Recruitment of Volunteers

Human volunteers were recruited among students in five agricultural colleges in Denmark. All volunteers participated in information meetings, received written project information, signed a declaration of informed consent, and filled in a questionnaire. Subjects were eligible for participation if they (1) were healthy individuals above 18 years of age, (2) had not visited swine farms 1 week before the study, (3) did not work in health care facilities, (4) did not have allergies to dust, (5) did not use antibiotics, (6) did not have skin diseases or wounds, and (7) were tested MRSA negative before the farm visit. Participants that had been in contact with MRSA-infected herds after the farm visit or found to harbor other *spa* types of MRSA than what was found in the farm were excluded from the follow-up investigation on days 1, 2, and 7.

The study was performed in accordance with principles of the Declaration of Helsinki and was approved by the National Committee on Health Research Ethics (Protocol H-15013814).

### Study Design

The trials were conducted in 2017 on five swine farms that were found LA-MRSA-positive prior to the farm visit. A total of 163 volunteers were included, 118 participated in the mask trials (units 1–5 in Tables 1, 2) and were randomly allocated to the group wearing masks and the control group. In addition, 45 volunteers were included to increase the precision of estimating a colonizing dose for humans relative to the concentration of airborne MRSA (units 6–8 in Table 2).

**Table 1:** Protective effect of dust mask on MRSA contamination of 118 volunteers after 1-h visit to MRSA-positive swine farms.

Unit	Farm	Mask	Day 0			Day 1			Day 2			Day 7		
						
			*n*	Pos	%	*n*	Pos	%	*n*	Pos	%	*n*	Pos	%
1	1	Yes	12	2	17	11	0	0	11	0	0	11	0	0
		No	13	11	85	10	2	20	10	0	0	9	0	0
2	2	Yes	17	0	0	16	0	0	16	0	0	13	0	0
		No	17	9	53	17	1	6	17	1	6	13	0	0
3	2	Yes	7	2	29	7	0	0	6	0	0	4	0	0
		No	9	3	33	9	0	0	9	2	22	3	0	0
4	3	Yes	10	0	0	10	0	0	10	0	0	10	0	0
		No	11	6	55	11	0	0	10	0	0	10	0	0
5	4	Yes	11	1	9	11	0	0	9	0	0	11	0	0
		No	11	9	82	11	2	18	11	2	18	9	1	11

Total		Yes	57	5	9	55	0	0	52	0	0	49	0	0
		No	61	38	62	58	5	9	57	5	9	44	1	2


**Table 2:** Methicillin-resistant *Staphylococcus aureus* (MRSA) concentration in air and MRSA contamination of 106 volunteers not wearing masks after 1 h visit to MRSA-positive swine farms.

Unit	Farm	MRSA air		Day 0			Day 1			Day 2			Day 7		
						
		CFU/m^3^	*SD*	*n*	Pos	%	*n*	Pos	%	*n*	Pos	%	*n*	Pos	%
1	1	517^1^	216	13	11	85	10	2	20	10	0	0	9	0	0
2	2	163	21	17	9	53	17	1	6	17	1	6	13	0	0
3	2	33	23	9	3	33	9	0	0	9	2	22	3	0	0
4	3	24	14	11	6	55	11	0	0	10	0	0	10	0	0
5	4	353	371	11	9	82	11	2	18	11	2	18	9	1	11
6	2	60	23	12	9	75	12	1	8	12	0	0	8	0	0
7	2	117	51	17	15	88	12	0	0	12	2	17	10	1	10
8	5	21	43	16	4	25	16	0	0	16	0	0	15	0	0

Total				106	66	62	98	6	6	97	7	7	77	2	3


*^1^Mean*.

### Procedures at the Farm

Before entering the farm, all volunteers washed their hands and changed clothes wearing a clean pair of boots and a disposable suit (Tyvek^®^ Classic Xpert, DuPont, United States) covering the whole body including the hair, leaving only the face, and hands exposed. Participants wearing masks entered the farm first and were also the first to leave the farm, and the nasal samples were taken immediately after removing the mask in the changing room. The masks used were P2 masks (3M FFP2 model 8822) designed to filter out airborne dust but not airborne microorganisms. During the visit, the participants stayed in the corridor separating the pens for 60 min. When leaving the farm, the volunteers changed clothes and washed their hands followed by disinfection with 70–85% ethanol.

### Human Sampling

Nasal samples were taken by rotating the eSwab^TM^ (Copan) five times in the anterior part of each nostil. Nasal samples were taken by the investigators 1 h before entering the farms (PRE samples) and immediately after leaving the farm (*T* = 0). All samples were kept at 4°C until cultivation next morning. Additional nasal samples were taken one (*T* = 1), two (*T* = 2), and 7 days (*T* = 7) after the visit by the volunteers and sent to the laboratory without cooling.

### Microbiological and Molecular Analyses of Human Samples

From each *T* = 0 sample, MRSA was quantified by making serial dilutions of the swab fluid (1 ml) with 0.9% NaCl added 0.1% Triton X-100 (Sigma-Aldrich) followed by spread of 100 μl on liance MRSA 2 agar plates (Oxoid) and incubation at 35°C for 22–24 h. Furthermore, all samples were investigated for MRSA by enrichment in tryptic soy broth (Sigma-Aldrich) supplemented with 6.5% NaCl at 35°C for 16–24 h followed by spread of 10 μl on Brilliance MRSA 2 agar plates and incubation at 35°C for 22–24 h. MRSA was identified as denim blue colonies. One colony from each volunteer at *T* = 0 was selected for molecular verification. In addition, one colony from each person showing growth of presumptive MRSA-colonies on day 1 or later was verified by PCR.

All MRSA subcultures were verified by a PCR assay detecting *mecA*, *lukF-PV*, *scn*, and *spa* followed by *spa* typing (Islam et al., 2017). MRSA was identified by the presence of *mecA* and *spa* amplicons.

### Sampling of Airborne MRSA

Airborne MRSA was sampled on gelatin filters using an AirPort MD8 air sampler (Sartorius, Germany) at a flow rate of 50 l/min. The air sampler was held at a height of approximately 150 cm. Between 100 and 500 l air was sampled to each gelatine filter where after the filter was transferred to a MRSA 2 agar plate (Oxoid) to which the filter adhered and dissolved. Air sampling was performed 3–11 times during the farm visit (Supplementary Table S1) and the different parts of the room were systematically sampled during the 1-h visit. The MRSA 2 agar plates were incubated approximately 3 h after leaving the farm at 35°C for 22–24 h. The number of colonies was counted and the air concentration of MRSA calculated. One presumptive MRSA-colony per agar plate was confirmed as MRSA as described above.

### Statistical Analysis

The results from the MRSA cultivation after enrichment (positive or negative) on samples obtained immediately after leaving the farm (*T* = 0) were used for the statistical analyses.

The risk of contamination of MRSA with respect to using a mask was estimated using logistic regression models with farm as random effect by the SAS GLIMMIX procedure. The models were adjusted for the following covariates obtained from the questionnaires: gender, age (continuous), and smoking habit (yes/no).

The risk of contamination of MRSA with respect to the amount of airborne MRSA was estimated using a joint model to take into account that the amount of airborne MRSA was measured several times within each unit. The joint model was a combination of logistic regression (proportion contaminated) and Poisson regression (airborne MRSA) and estimated in the SAS NLMIXED procedure using Newton-Rapson with ridging technique.

All statistical analyses were performed using SAS Version 9.4 (SAS Institute Inc., United States).

## Results

### General Results

There were 180 volunteers from five agricultural colleges. Seventeen volunteers (9%) were excluded due to being MRSA-positive before entering the farm, giving a total of 163 participants. Of the 118 volunteers participating in the mask trials, 57 (48%) carried a dust mask during the farm visit (Table 1). In addition, 45 volunteers participated in trials not involving the use of dust masks; in total 106 persons visited the farms without wearing mask protection (Table 2).

Eight units/rooms on five swine farms were visited and the number of participants per unit varied between 12 and 34 (Tables 1, 2). The average age of the participants was 26 years (range 18–68, *SD* = 13), 73% were male and 28% smoked daily. The mean air concentration of MRSA varied between 21 and 517 CFU/m^3^ (Table 2).

The nasal load of MRSA was generally very low (1–2 colonies/agar plate or only detectable after enrichment) and the quantitative data was not regarded as suitable for further investigations. The following presentation is therefore based on qualitative data, obtained after enrichment of nasal samples. In all herds, only MRSA CC398 *spa* type t034 was found.

### Protective Effect of Using Dust Mask

After leaving the farm, 9% of the participants wearing masks were MRSA-positive (range 0–29% between farms) compared to 62% of the participants not wearing masks (range 33–85%) (Table 1). At day 1, 2, and 7, none of those wearing masks were MRSA-positive, whereas, 9% among the participants not wearing masks were still MRSA-positive after 1 and 2 days and one participant also after 7 days (Table 1).

A clear protective effect of wearing mask was observed in the statistical analyses. An unadjusted odds ratio of 18.9 (CI: 6.4–56.2) for being MRSA-positive for those not wearing masks compared to those wearing masks was found when including the farm as random effect. None of the covariates (gender, age, and smoking) had any major effect on the risk for being MRSA-positive (*p* > 0.55) and did not influence the risk for not using mask (OR 18.3; CI: 6.1–54.8).

### Contamination Dose

**FIGURE 1:**
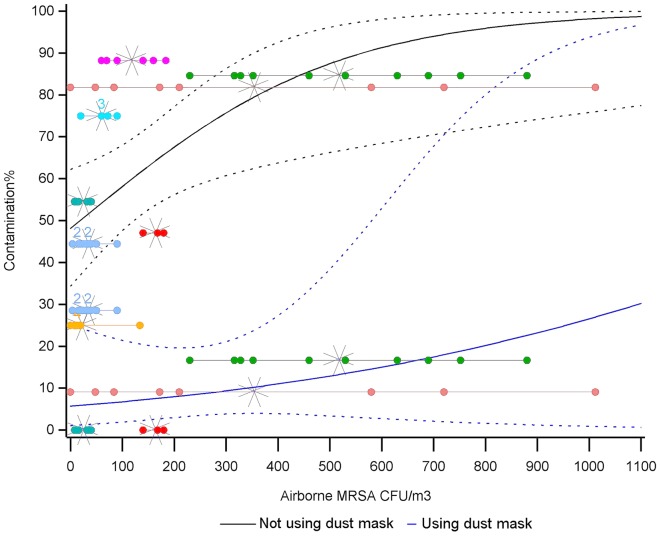
Plot showing the correlation between the amount of airborne MRSA (CFU/m^3^) and the fraction of volunteers being contaminated by MRSA after an 1-h visit to a MRSA-positive swine farm. The upper curve (solid) shows the point estimates and confidence limits (dotted lines) for the 106 participants not wearing masks. The lower curves show the corresponding lines for the 57 participants wearing dust masks. The horizontal lines show the different measurements of airborne MRSA within the eight swine units. Measurements within the same unit share colors, a number indicates when a dot represents more than one measurement, and the cross shows the mean of the measurements in the unit.

An attempt was made to obtain an estimate of the contamination dose of airborne MRSA based on the 106 participants not wearing masks (Table 2). Due to the high variation in the MRSA measurements (Figure 1 and Supplementary Table S1), it was not possible to establish a stable estimate for this. To illustrate the instability of the estimation, a bootstrap analysis on unit level was performed (Supplementary Figure S1), showing that a 50% contamination dose lay in the range of 20–90 CFU airborne MRSA/m^3^ and when excluding unit 8 an 50% contamination dose could not be estimated. As all curves have to go through the origo, the curves in the lower range of airborne MRSA will necessarily be very steep. The lower curve (Figure 1) shows the point estimates and confidence intervals for the 57 participants wearing P2-masks. With an airborne amount of 300 CFU MRSA/m^3^, approximately 10% of those wearing masks were MRSA-positive (CI: 4–23%).

## Discussion

### Protection Against MRSA Contamination

Dust masks showed a clear protective effect during the farm visits. There was a highly increased probability (OR = 18.9) for becoming MRSA-positive for the volunteers that were not wearing masks compared to those wearing masks. Dust masks are not designed to filter out single bacterial cells so the protective effect must at least partly reflect that MRSA is associated to bigger dust particles (Zhao et al., 2017; Feld et al., 2018). Interestingly, Barnhart et al. (1997) reported a 17.5-fold protection against *Mycobacterium tuberculosis* when using a dust-mist mask compared to not using masks. Our study confirms the findings of Nathaus et al. (2011), where four out of five veterinarians wearing P2-masks during all farm visits were still MRSA-negative after 30 days, even though 38% of the farms were MRSA-positive during this period. Furthermore, P2 dust masks may protect against airway inflammation following exposure in a swine barn, as shown by Sundblad et al. (2002).

Mask efficiency is mainly determined by three factors: filter efficiency, leakage though valves and valve seats, and leakage between face and mask (Brosseau et al., 1993). In the present investigation, 9% of the volunteers using masks were MRSA-positive when leaving the farm. The reason for this could be a combination of all these three factors but could also be due to contamination in connection with mask removal and sampling.

### Duration of MRSA Contamination

Among the 106 volunteers not wearing masks, 62% were found MRSA-positive when leaving the farm (Table 2). In an earlier investigation, 94% of the volunteers were found MRSA-positive after a 1-h visit to a swine farm (Angen et al., 2017). The difference in contamination levels can probably be explained by differences in the average level of airborne MRSA in the two studies (geometric mean: 88 vs. 384 CFU MRSA/m^3^). Out of the 106 volunteers, 6, 7, and 3% tested MRSA-positive 1, 2, and 7 days after the farm visit, respectively. This corresponds closely to the duration of MRSA positivity reported by Angen et al. (2017), being 11, 6, and 1%, respectively. Apparently, a few visitors may retain MRSA in the nose for a few days and up to a week after a short-time visit to an MRSA-positive farm. A follow-up investigation of the two participants being MRSA-positive on day 7 was not possible, as they had reentered swine farms as part of their education. The initial screening of the students found that 9% were MRSA-positive, most of these were students that regularly worked with pigs. One can speculate if these persons also had regular work with pigs before the studies and thereby could be more prone to be contaminated for longer times, e.g., due to a changed nasal microbiome.

### Quantification of Airborne MRSA

A number of sampling methods have been applied for quantification of airborne microorganisms, e.g., impaction, impingement, and filtration (Grinshpun et al., 2016). Filtration sampling is a commonly used method but may result in loss of viability due to desiccation stress during sampling. In this investigation, we tried to minimize the desiccation stress by using short-term sampling on to a gelatine filter followed by incubating directly on an MRSA selective agar. This method has earlier been reported to have good efficiency (Burton et al., 2007; Zhao et al., 2011). This method made it possible to achieve a low detection limit for MRSA (2 or 10 CFU MRSA/m^3^ when sampling 0.5 or 0.1 m^3^ air, respectively). In the present investigation, we used this in an attempt to estimate the airborne MRSA level where 50% of the volunteers were contaminated (CON_50_). However, there was a high variation in the measurements of airborne MRSA related to position in the stable, and time during the stay. This might reflect different dust levels due to animal activity and position relative to the ventilation system. In addition, the bootstrap analysis showed that the estimate was highly dependent on inclusion or exclusion of single experiments (Supplementary Figure S1). As all curves have to go through the origo, the curves will necessarily be very steep for low MRSA values. It was therefore not possible to establish a stable estimate for this CON_50_. Although MRSA could be cultivated from a few volunteers up to 7 days after the farm visit, none of the volunteers should be considered as having been colonized by MRSA, which is in accordance with what was reported by Angen et al. (2017). A tentative conclusion is that human colonization is dependent on repeated exposure to LA-MRSA over extended time periods and will not be observable in short-term investigations. To determine an colonizing dose for LA-MRSA might be of public health significance, but will probably be very difficult to estimate, both of ethical reasons and because LA-MRSA has been shown to have limited infective potential compared to more human-adapted lineages of staphylococci (Köck et al., 2011).

### Public Health Significance

This study shows that the use of dust masks gives a significant protection against nasal MRSA contamination after a short-term visit to a swine farm. Due to the rapid decline in the nasal MRSA levels after a farm visit and the transient nature of the contamination (Angen et al., 2017), the risk for short-term visitors to cause secondary transmissions of MRSA is most likely negligible and the public health benefit of using masks for short-term visitors is probably limited. However, other groups that have longer and more frequent contact to swine farms have higher probability for being colonized by LA-MRSA, e.g., farmers, veterinarians, and craftsmen. In this connection it is noteworthy that the Danish Working Environment Authority limits the use of dust masks to a maximum of 3 h per day. The use of dust masks against MRSA colonization has earlier been demonstrated for a small group of veterinarians (Nathaus et al., 2011) and a more thorough evaluation of the benefit for this group of farm visitors is recommendable. The use of dust masks could give personal protection against colonization or infection, reduce secondary transmission to the society, but also protect against human transmission between swine herds. In connection with an attempt to eradicate LA-MRSA from swine herds, which has been done in Norway (Grøntvedt et al., 2017), the protection of the swine herds against LA-MRSA carried by humans has turned out to be of great importance.

## Author Contributions

ØA, PB, and AL designed the study. ØA, LS, and TU performed the field trials. MA performed the statistical analyses. ØA drafted the manuscript. All authors have approved the final version of the manuscript.

## Conflict of Interest Statement

The authors declare that the research was conducted in the absence of any commercial or financial relationships that could be construed as a potential conflict of interest.
