# Supplementary material for: Controlling Transmission of MRSA to Humans During Short-Term Visits to Swine Farms Using Dust Masks
Source: Front Microbiol. 2019 Jan 17;9:3361. doi: 10.3389/fmicb.2018.03361 (PMC6345077; doi:10.3389/fmicb.2018.03361)

Supplementary Material

Controlling transmission of MRSA to humans during short-term visits to swine farms using dust masks

Øystein Angen*, Lotte Skade, Tinna Ravnholt Urth, Mikael Andersson, Poul Bækbo, Anders Rhod Larsen

*** Correspondence:** Øystein Angen: ysan@ssi.dk

Supplementary Table (1) and Figure (1)

Supplementary Table S1. Measurements of airborne MRSA in 8 swine units

Unit	Farm	Measurements	Mean[1] (sd)	Median (IQ)[2]	Min-Max
1	1	10	517 (216)	495 (328-690)	230-880
2	2	3	163 (21)	168 (140-180)	140-180
3	2	11	33 (23)	30 (16-40)	4-90
4	3	6	24 (14)	24 (12-38)	8-40
5	4	8	353 (371)	191 (66-650)	0-1012
6	2	6	60 (23)	60 (60-72)	20-90
7	2	6	117 (51)	115 (70-160)	60-184
8	5	9	21 (43)	8 (0-18)	0-134

[1] CFU MRSA/m^3

[2] Interquartile range

Supplementary Figure S1.

Bootstrap analysis of the correlation between the amount of airborne MRSA (CFU/m^3) and the fraction of volunteers beeing contaminated by MRSA after an one-hour visit to MRSA-positive swine farms. Each line represents the point estimates when including all units (thick black line) or when excluding one of the eight units (different colored lines). The horizontal line indicates that 50% of the participants were MRSA contaminated. The light blue line does not cross the 50% line and shows the point estimates when excluding unit 8 from the analysis.

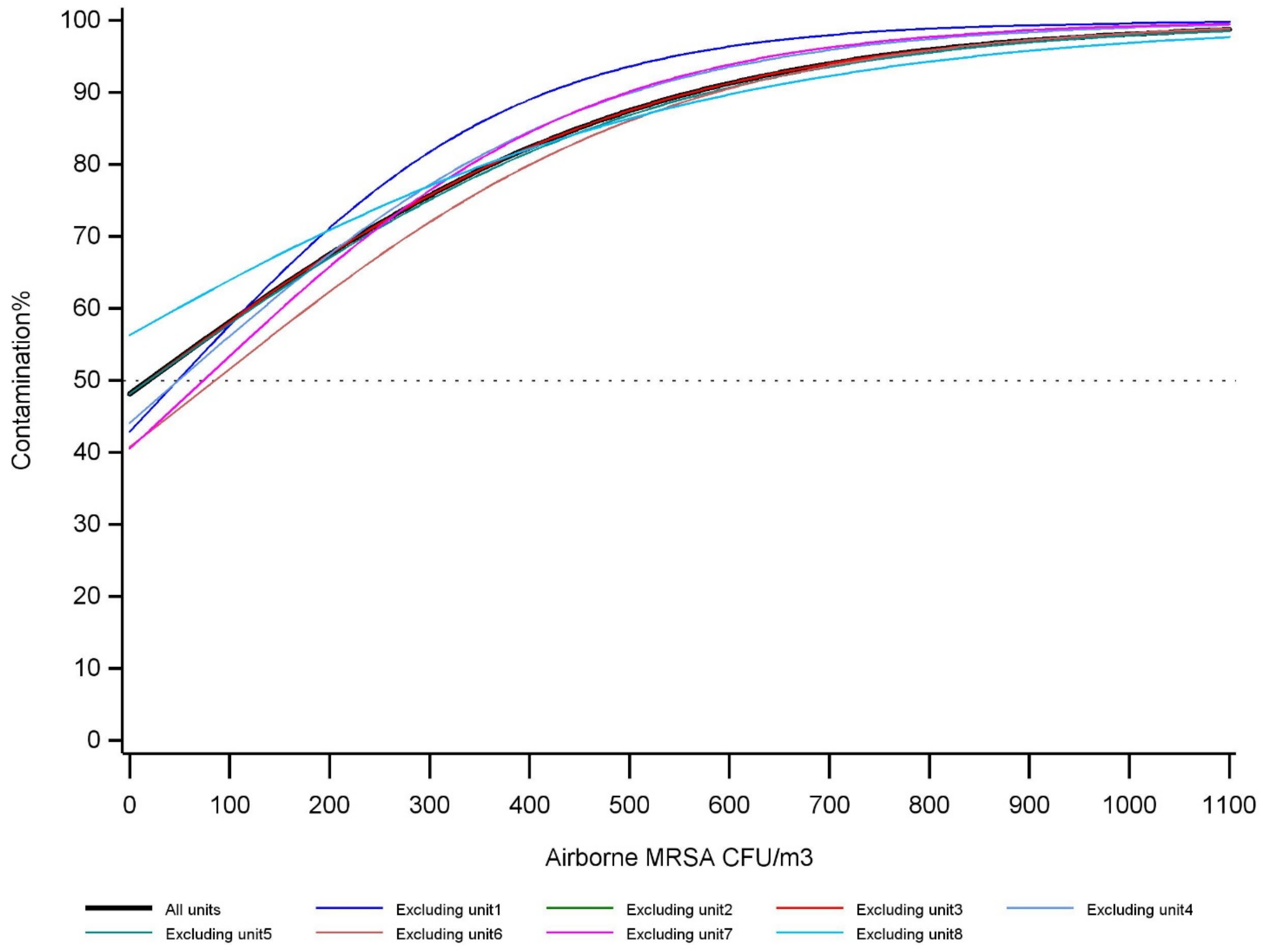

Supplement: Supplementary file 1 [file Data_Sheet_1.pdf]
